# Delving Into the Interaction Between Exercise and Diabetes on Methylation of the FKBP5 Gene

**DOI:** 10.1155/jdr/1162708

**Published:** 2025-02-19

**Authors:** Teng-Chi Yang, Jen Pi Tsai, Honda Hsu, Yen-Chung Chen, Yi-Chia Liaw, Shu Yi Hsu, Hao Jan Yang, Yung-Po Liaw

**Affiliations:** ^1^Department of Public Health and Institute of Public Health, Chung Shan Medical University, Taichung City, Taiwan; ^2^Department of Medical Affairs, Dalin Tzu Chi Hospital, Buddhist Tzu Chi Medical Foundation, Chiayi County, Taiwan; ^3^Division of Nephrology, Dalin Tzu Chi Hospital, Buddhist Tzu Chi Medical Foundation, Chiayi County, Taiwan; ^4^School of Medicine, Tzu Chi University, Buddhist Tzu Chi School Foundation, Hualien County, Taiwan; ^5^Division of Plastic Surgery, Dalin Tzu Chi Hospital, Buddhist Tzu Chi Medical Foundation, Chiayi County, Taiwan; ^6^Department of Neurology, Changhua Christian Hospital, Changhua City, Taiwan; ^7^Neurological Institute, Taipei Veterans General Hospital, Taipei City, Taiwan; ^8^Institute of Medicine, Chug Shan Medical University, Taichung City, Taiwan; ^9^Department of Medical Imaging, Chung Shan Medical University Hospital, Taichung City, Taiwan

**Keywords:** cg00862770, cg22363520, diabetes mellitus (DM), DNA methylation, exercise, FKBP5 gene

## Abstract

**Objective:** FKBP5 is a critical gene involved in regulating the hypothalamic–pituitary–adrenal (HPA) axis and stress response. Aberrant DNA methylation at FKBP5 cytosine–phosphate–guanine (CpG) sites, such as cg22363520 and cg00862770, has been implicated in mental health disorders and metabolic diseases, including Type 2 diabetes. Exercise is a modulator of DNA methylation and metabolic health. This study investigates the interaction between exercise, diabetes, and FKBP5 methylation at cg22363520 and cg00862770 and explores their implications for mental health and disease development.

**Materials and Methods:** FKBP5 methylation levels at cg22363520 and cg00862770 were analyzed in a cohort stratified by diabetes and exercise. Multiple linear regression models assessed the main effects and interactions of exercise and diabetes on FKBP5 methylation, with further stratified analyses for site-specific effects.

**Results:** Exercise and diabetes showed significant and site-specific effects on FKBP5 methylation at cg22363520 and cg00862770. At cg22363520, exercise significantly reduced methylation levels in nondiabetic participants (*β* = −0.00195, *p* = 0.0157), while no significant effect was observed in diabetic individuals. Conversely, at cg00862770, exercise significantly decreased methylation levels in diabetic participants (*β* = −0.00611, *p* = 0.0081), with no significant effect in the nondiabetic group. Diabetes itself was associated with increased FKBP5 methylation at both sites, particularly in individuals without regular exercise. Additionally, significant interaction effects between exercise and diabetes were identified for both cg22363520 (*p* = 0.0336) and cg00862770 (*p* = 0.0021), highlighting the interplay between metabolic status and physical activity in regulating FKBP5 methylation.

**Conclusion:** This study demonstrates that the effects of exercise on FKBP5 methylation are site-specific and influenced by diabetes status. Exercise reduces methylation at cg22363520 in nondiabetics and at cg00862770 in diabetics, indicating its role in modulating epigenetic regulation of stress and metabolic pathways. These findings underscore the interplay between exercise, diabetes, and FKBP5 methylation, with potential implications for improving mental health and metabolic outcomes.

## 1. Introduction

The number of diabetes patients increased from 108 million in 1980 to 422 million in 2014. From 2000 to 2019, the age-standardized mortality rate for diabetes increased by 3%. According to the latest data from the International Diabetes Federation (IDF), as of 2021, the actual number of individuals affected is close to 537 million.

There are many reasons for the increase in diabetes, these include poor dietary habits, obesity, decreased physical activity, lack of exercise, lifestyle stresses, and genetic factors [1–3].

There is a certain correlation between diabetes and stress and immunity. Long-term stress can lead to the release of stress hormones such as cortisol and adrenaline, affecting the action of insulin and leading to elevated blood sugar levels [4]. Chronic stress is associated with an increased risk of Type 2 diabetes (T2D). Oxidative stress and inflammation are potential mediators of this risk and can increase the risk of newly diagnosed patients developing T2D [5]. Furthermore, T2D is an inflammatory cytokine–related disease and high blood sugar levels may affect the normal function of the immune system, making the body incapable of resisting attacks from viruses and bacteria [6]. Additionally, diabetic patients are more prone to chronic diseases, further weakening their immunity. Furthermore, diabetes is one of the leading causes of blindness, renal failure, heart disease, stroke, and lower limb amputation [7]. Improving blood sugar control in patients with T2D can prevent complications and reduce the incidence and mortality of related diseases [8, 9]. Therefore, diabetes mellitus (DM) is one of the most serious health problems today, which not only affects patients but also has serious implications for our society.

Studies have shown that exercise has significant benefits for patients with diabetes [10]. The exercise intervention leads to clinically significant improvements in blood sugar control compared to the control group [11], and participants who exercise regularly maintain improved blood sugar control [12]. In summary, physical training has a beneficial effect on the treatment of diabetes. Exercise plays a role in the regulation of glucose transport during exercise, effectively improving glucose metabolism and playing an important role in the prevention and treatment of diabetes [13–15].

The FKBP5 gene encodes a protein that plays an important role in physiology and nervous system functions, including stress response [16–18] and immune regulation [19]. Previous research has indicated that DNA methylation of the FKBP5 gene can be regulated by stress and aging and is associated with increased cardiovascular risk [20, 21].

It functions as a regulator of glucocorticoid (GC) signaling, and the FKBP5 gene is associated with many stress-related psychiatric disorders. These include bipolar disorder, depression, anxiety disorder, and PTSD [22, 23]. Given the role of FKBP5 in regulating the stress response system and its potential impact on psychiatric disorders, genetic variations within the gene can influence hippocampal structure and function differently, as well as regulate the sensitivity of the glucocorticoid receptor (GR) and hypothalamic–pituitary–adrenal (HPA) axis responsiveness. These disorders include bipolar disorder, depression, anxiety disorders, and posttraumatic stress disorder [21, 23–26].

Overexpression of FKBP5 may lead to GR dysfunction, disrupting the negative feedback regulation of the HPA axis. This could be a key link between stress and psychiatric disorders [27]. Elevated FKBP5 expression is associated with insulin resistance, impaired glucose metabolism, and increased inflammation levels, all of which are characteristic features of T2D [28, 29]. Overall, FKBP5 plays a pivotal role in stress response, immune regulation, and metabolic pathways. Dysregulation of FKBP5 expression has been linked to stress-related psychiatric disorders, inflammation, and metabolic diseases.

DNA methylation at cytosine–phosphate–guanine (CpG) sites is a critical epigenetic mechanism regulating gene expression. Methylation typically suppresses gene activity when present in promoter regions, while hypomethylation can increase transcription. CpG sites act as molecular switches, allowing dynamic adaptation to environmental and physiological stimuli. The methylation status of FKBP5-specific CpG sites can modulate GR sensitivity, linking stress and metabolic responses to changes in gene expression [25–27].

This study focuses on two key CpG sites, cg22363520 and cg00862770, due to their regulatory roles in FKBP5 expression and stress response and their relevance to the metabolic disease diabetes [30].

Abnormal methylation of cg22363520 is closely associated with elevated FKBP5 expression, particularly impacting the regulatory function of the HPA axis in the context of stress. Studies have shown that GC exposure can lead to changes in FKBP5 methylation, further disrupting glucose metabolism and exacerbating insulin resistance. These changes may persist for years, resulting in long-term effects on metabolic health [31].

The cg22363520 site is located in the regulatory region of FKBP5 and can influence the sensitivity of the GR. Hypomethylation of cg22363520 enhances FKBP5 expression, amplifies stress responses, disrupts glucose metabolism, and promotes insulin resistance [32]. Studies have shown that its demethylation, in the context of stress, may be associated with metabolic health outcomes, including diabetes [33, 34]. The literature indicates that the DNA methylation patterns of the FKBP5 gene undergo significant changes from childhood to adolescence, suggesting its critical role in stress response and metabolic function regulation [35]. The potential role of cg22363520 in regulating inflammatory processes suggests that it may contribute to diabetes development through mechanisms involving insulin resistance and *β*-cell dysfunction [36, 37].

The cg00862770 site is associated with the regulation of adipocyte function and glucose homeostasis by FKBP5. Increased methylation of cg00862770 is linked to reduced FKBP5 expression, impaired glucose uptake, and exacerbation of diabetes-related metabolic dysfunction [28, 29]. Studies suggest that DNA methylation at this site may influence susceptibility to T2D through its effects on the expression of genes involved in metabolic processes [38, 39]. Additionally, studies indicate that DNA methylation at this site can affect the expression of genes involved in metabolic pathways, potentially influencing insulin sensitivity and secretion [40]. Furthermore, T2D is characterized by insulin resistance and insufficient insulin secretion by pancreatic *β*-cells. The methylation status of cg00862770 may promote these processes by regulating FKBP5 expression. FKBP5 influences the body's responses to stress and inflammation, which are known to exacerbate insulin resistance [41].

There is also evidence that methylation differences at cg00862770 are not only associated with diabetes but may also be linked to other conditions, such as depression and stress-related disorders. These associations suggest a broader role for FKBP5 in mediating responses to environmental stressors, which may indirectly impact metabolic health [39]. In other studies, GC therapy leads to increased FKBP5 methylation levels, which subsequently affect glucose metabolism and disrupt adipocyte function [42].

The methylation levels of cg00862770 have been associated with patients' psychopathological features, such as fatigue, depression, or anxiety. This suggests that methylation changes at this site may reflect the impact of endogenous hypercortisolism on gene regulation [43].

Recent studies have begun to explore a possible connection between methylation of the FKBP5 gene and diabetes [29]. The FKBP5 gene in human subcutaneous adipose tissue (SAT) tends to increase in T2D subjects and is associated with elevated glucose levels [28]. Exercise is known to modulate DNA methylation at genes associated with metabolism and stress. Interventional studies have confirmed significant effects of acute or chronic exercise on DNA methylation in different tissues. The hypomethylation of cg22363520 after exercise may enhance GR sensitivity and reduce stress-induced inflammation. Similarly, methylation changes in cg00862770 may promote glucose metabolism, further supporting the critical role of exercise in diabetes prevention and management [24, 28, 44].

Additionally, interventional studies have shown that acute or chronic physical activity can reverse adverse methylation patterns at FKBP5 CpG sites, including cg22363520. This process may improve stress adaptation and metabolic outcomes by normalizing FKBP5 expression [24, 29, 44]. The dynamic methylation changes induced by exercise provide a potential therapeutic mechanism to mitigate diabetes-related epigenetic alterations.

In summary, the function of the FKBP5 gene is closely related to stress response and immune regulation. Recent research also suggests that methylation of the FKBP5 gene may be related to mental health, stress response, and diabetes. Furthermore, exercise, as an important lifestyle intervention, plays a key role in the prevention and management of diabetes. However, more research is still needed to fully understand whether exercise and diabetes have regulatory effects and effects on DNA methylation of the FKBP5 gene. Therefore, the purpose of this article is to explore the potential relationship between exercise, diabetes, and methylation of the FKBP5 gene.

## 2. Materials and Methods

### 2.1. Data Source

The study is aimed at investigating the interaction between diabetes and exercise on FKBP5 methylation at the methylation of the cg22363520 and cg00862770 loci in individuals aged 30–70, who are Taiwanese citizens, without foreign bloodlines and without a history of cancer diagnosis. Data for the study were obtained from the Taiwan Biobank database, which currently contains data from approximately 109,747 residents. Informed consent was obtained from all participants prior to data collection. DNA methylation evaluation was performed on DNA extracted from blood samples using the Infinium MethylationEPIC BeadChipEPIC array (Illumina Inc.).

### 2.2. Study Participants

A total of 1103 individuals (562 men, 541 women) were included as study participants. The demographic data retrieved from the database included age, sex, body fat rate, waist–hip ratio, and lifestyle factors (regular exercise, smoking, second-hand smoke, and alcohol consumption), and the genetic information recovered was cg22363520 and cg00862770 on the FKBP5 gene.

The CpG sites cg22363520 and cg00862770 are specific DNA methylation loci within the FKBP5 gene. These sites are known to modulate FKBP5 gene expression, influencing stress response and immune regulation. Previous studies have linked cg22363520 to GR sensitivity and stress-related psychiatric disorders, while cg00862770 has been associated with transcriptional regulation of FKBP5 in metabolic and stress-related conditions.

The definition of exercise was defined as “no” indicating not meeting the regular exercise. “yes” indicated physical activity of at least three times a week lasting at least 30 min each session. Regarding smoking, “never” indicated individuals had never smoked or had not smoked for more than 6 months, “quit” referred to those who had already quit smoking or rarely smoked, and “current” indicated individuals who had smoked for over 6 months and continued to do so. For alcohol consumption, “never” indicated individuals who had never consumed or did not consume more than 150 cc per week, “quit” referred to those who had already quit alcohol consumption for at least 6 months, and “current” indicated individuals who consistently consumed 150 cc per week for at least 6 months.

### 2.3. Statistical Analysis

SAS 9.4 software was used for statistical analysis. Chi-square tests, *t*-tests, and multivariate linear regression models were used to assess the interaction between diabetes and exercise on methylation levels. Data were managed and analyzed using SAS 9.4 software (SAS Institute, Cary, North Carolina).Chi-square tests were used to compare the percentages of diabetes and categorical variables. *T*-tests were used to compare the mean continuous variables on participants with and without diabetes. Multivariate linear regression models were used to determine the interaction between diabetes and exercise habit on methylation levels (cg22363520 and cg00862770 in the FKBP5 gene). Data were presented as numbers (percentages) for categorical variables and mean ± standard error for continuous variables.

## 3. Result

Table 1 presents the baseline characteristics of participants, stratified by diabetes status, including age, gender, body fat, waist-to-hip ratio, smoking status, and methylation levels. Among the 1103 participants recruited, 105 had diabetes. Diabetic participants were significantly older than nondiabetic participants (55.99 ± 0.93 years vs. 48.96 ± 0.35 years, *p* < 0.0001) and had a higher waist-to-hip ratio (0.9208 ± 0.00601 vs. 0.8599 ± 0.00205, *p* < 0.0001), indicating a greater proportion of central obesity among diabetic individuals. Additionally, data stratified by gender and exercise status are provide in Tables S1-1 and S1-2, including body fat percentage and waist-to-hip ratio.

Interestingly, the proportion of regular exercisers was higher among diabetic participants compared to nondiabetic participants (58.10% vs. 42.59%, *p* = 0.0023). This study analyzed DNA methylation levels of the FKBP5 gene (cg22363520 and cg00862770) in both diabetic and nondiabetic participants. The mean ± standard error of cg22363520 methylation in diabetic participants was 0.9147 ± 0.00119, compared to 0.9142 ± 0.000386 in nondiabetic participants. For cg00862770, the mean ± standard error was 0.0512 ± 0.00108 in diabetic participants and 0.0507 ± 0.000349 in nondiabetic participants.

Although the methylation levels of cg22363520 and cg00862770 were slightly higher in diabetic participants, the differences were not statistically significant, warranting further investigation.

Table 2 shows the results of the interaction between diabetes and exercise on FKBP5 methylation levels (cg22363520 and cg00862770). Both interactions were found to be significant, with *p* values of 0.0336 and 0.0021, respectively. The effects of diabetes and exercise on cg22363520 methylation levels were *β* = 0.00125 (*p* = 0.3202) and −0.00144 (*p* = 0.0590), respectively. Similarly, the effects of diabetes and exercise on methylation levels were *β* = 0.00047263 (*p* = 0.6875) and −0.00020538 (*p* = 0.7736), respectively. Furthermore, age was found to be significant in both the cg22363520 and cg00862770 model (*β* = 0.00014311, *p* = 0.0001, and *β* = −0.00011592, *p* = 0.0008, respectively).

The *β* value quantifies the relationship between these factors (e.g., diabetes and exercise) and the methylation levels at specific CpG sites (cg22363520 and cg00862770). The size of the *β* value reflects the strength of the effect, with larger absolute values indicating a more significant impact.

In Table 3, based on the results of the interaction analysis, we further conducted a stratified analysis by diabetes status to investigate the effect of exercise on FKBP5 methylation at the cg22363520 and cg00862770 loci. This stratified analysis highlighted the potential interactions among diabetes, exercise, and FKBP5 methylation, considering the metabolic and inflammatory changes associated with diabetes. For cg22363520, regular exercise habits significantly reduced methylation levels in nondiabetic participants (*β* = −0.00195, *p* = 0.0157). Similarly, regular exercise habits also significantly reduced methylation levels in diabetic participants (*β* = −0.00611, *p* = 0.0081). The findings reveal the differential effects of exercise on FKBP5 methylation in diabetic and nondiabetic participants, emphasizing the importance of site-specific methylation changes.

At the cg22363520 site, the negative *β* value for exercise indicates that regular exercise reduces the gene's methylation levels, potentially promoting gene expression and improving stress regulation. Conversely, the positive *β* value for diabetes suggests an association with increased methylation at this site, which may result in reduced gene expression and further impact stress-related physiological responses.

At the cg00862770 site, the positive *β* value for diabetes indicates that patients with a lack of exercise experience increased methylation levels, which are associated with metabolic abnormalities. Meanwhile, the negative *β* value for exercise suggests a protective effect, as it lowers methylation levels at this site in diabetic patients.

Furthermore, we further stratification by exercise in Table 4. The methylation levels of cg22363520 in participants with diabetes were significantly associated with regular exercise habits (*β* = 0.00377 and *p* = 0.0241), while significant results indicated that the methylation levels of cg00862770 were significantly associated with people without regular exercise habits (*β* = 0.00456 and *p* = 0.0076).

Finally, Figures S1-1, S1-2, and S1-3 summarize the mean methylation levels of FKBP5 at cg22363520 and cg00862770, presenting methylation data stratified by diabetes status, gender, and exercise status. Figure S1-4 presents a summarize model illustrating the relationship and interaction between exercise and diabetes concerning FKBP5 gene methylation levels at the two specific CpG sites (cg22363520 and cg00862770).

## 4. Discussion

To our knowledge, this is the first cohort study investigating the association between DNA methylation of the FKBP5 gene (cg22363520 and cg00862770) and diabetes and exercise using data from Taiwan Biobank and clinical research databases. Our findings demonstrate a significant interaction between exercise and diabetes on DNA methylation at specific CpG sites, highlighting the role of epigenetics in metabolic regulation and stress adaptation.

This study identified two key findings: (1) habitual exercise significantly reduces methylation levels of cg22363520 in nondiabetic participants and cg00862770 in diabetic participants, and (2) methylation at cg22363520 is significantly associated with exercise habits in diabetic individuals, while cg00862770 methylation correlates with infrequent exercise, highlighting site-specific epigenetic responses to lifestyle factors.

Diabetes is a chronic disease characterized by impaired insulin regulation and elevated blood sugar levels, leading to severe complications such as cardiovascular disease, renal failure, and neuropathy. Previous research underscores the importance of exercise in improving glycemic control and reducing cardiovascular risks [45–47]. Regular physical activity reduces adiposity, enhances insulin sensitivity, and improves endothelial function [48–50]. Moreover, exercise has broader physiological and psychological benefits, including improved lipid profiles, muscle strength, and reduced daily insulin requirements [13]. These effects support the use of exercise as a first-line intervention in diabetes management [45].

The FKBP5 gene encodes an immunophilin protein involved in stress regulation, immune function, and protein transport. FKBP5 modulates GR sensitivity, linking it to stress-related disorders, including PTSD and metabolic diseases [51–55]. Elevated FKBP5 expression has been observed in T2D patients and is associated with increased blood glucose levels, implicating it in metabolic dysfunction [28, 56]. Polymorphisms in FKBP5 are also associated with insulin resistance and obesity, further connecting this gene to metabolic diseases [57].

DNA methylation is a dynamic epigenetic mechanism regulating gene expression. Hypomethylation at FKBP5 CpG sites has been linked to increased gene expression, while hypermethylation suppresses activity [58–60]. FKBP5 methylation levels are influenced by environmental and physiological factors, such as stress and inflammation, and are associated with accelerated aging and cardiometabolic risks [60, 61]. For example, methylation changes at FKBP5 loci have been linked to stress-related psychiatric disorders, metabolic diseases, and immune-inflammatory conditions [29, 30, 62, 63].

Our focus on cg22363520 and cg00862770 stems from their regulatory roles in FKBP5 gene expression. cg22363520 is located in a regulatory region of FKBP5, and hypomethylation at this site increases FKBP5 expression, potentially amplifying stress responses and impairing glucose metabolism [27, 64]. On the other hand, methylation changes at cg00862770 influence adipocyte function and glucose homeostasis. Increased methylation correlates with reduced FKBP5 expression and impaired metabolic regulation in diabetic patients [28, 65]. These site-specific patterns highlight the gene's dual role in stress response and metabolic pathways.

Exercise-induced DNA methylation changes have been well-documented, particularly in genes associated with metabolism and stress [44, 66]. Our study reveals that regular exercise reduces cg22363520 methylation in nondiabetic individuals, improving stress regulation, while exercise decreases cg00862770 methylation in diabetic individuals, enhancing glucose metabolism. These findings align with studies demonstrating that exercise can alter DNA methylation patterns in skeletal muscle and adipose tissue, potentially mitigating diabetes risk and improving metabolic outcomes [67–71].

Our results suggest that diabetes modifies the relationship between exercise and FKBP5 methylation. Specifically, diabetes significantly influences cg22363520 methylation patterns in individuals who exercise. For cg00862770, diabetic participants who exercised showed reduced methylation levels, highlighting a site-specific response. These findings suggest that FKBP5 methylation patterns are modulated by diabetes-related metabolic dysfunction, although the exact mechanisms require further exploration.

The differential methylation effects observed at cg22363520 and cg00862770 underscore the complexity of FKBP5's regulatory mechanisms. Exercise emerges as a potential epigenetic modulator, offering a noninvasive strategy to improve metabolic health. However, the site-specific responses suggest that tailored interventions may be necessary to optimize outcomes. Future studies should investigate the functional significance of FKBP5 methylation changes in different tissues; explore long-term effects of exercise on FKBP5 methylation in diverse populations; and examine interactions between FKBP5 methylation, other genetic factors, and environmental influences.

The FKBP5 gene has been shown to be closely associated with stress responses and mental health disorders, such as depression and anxiety. The effects of diabetes and exercise on FKBP5 gene methylation observed in this study may further support psychological health interventions. The cg22363520 and cg00862770 loci within the FKBP5 gene could serve as biomarkers for evaluating the effectiveness of exercise interventions in diabetes management. Regular monitoring of these loci may help to understand stress regulation and metabolic improvement in clinical settings.

Our findings underscore the importance of exercise in modulating FKBP5 methylation levels and its associated risks. This highlights the role of lifestyle interventions in preventing the onset and progression of diabetes and its complications.

Future studies could validate these findings in larger and more diverse populations and assess the long-term clinical outcomes of modulating FKBP5 methylation through exercise and other interventions. Furthermore, incorporating FKBP5 methylation analysis into routine clinical workflows could bridge the gap between research and practice, thereby improving patient outcomes.

Despite these strengths, our study has several limitations. The findings may not generalize across all populations due to the specific demographic composition of the sample. Additionally, the cross-sectional design limits causal inferences about the relationship between exercise, diabetes, and FKBP5 methylation. Finally, the study relied on blood-derived DNA for methylation analysis, which may not fully capture tissue-specific epigenetic changes.

In conclusion, this study highlights the interplay between exercise, diabetes, and FKBP5 methylation at key CpG sites. Regular exercise influences FKBP5 methylation patterns, potentially mitigating metabolic dysfunction. However, diabetes modifies these epigenetic effects, suggesting a complex relationship between metabolic health, lifestyle factors, and epigenetic regulation. These findings provide a direction for personalized interventions targeting epigenetic mechanisms to promote metabolic health and prevent diabetes-related complications.

## 5. Conclusion

Our research results indicate that participants who exercise and have diabetes are more likely to have methylation at the cg22363520 locus of the FKBP5 gene compared to those who exercise but do not have diabetes, and this gene is less likely to be expressed. For participants who do not exercise but have diabetes, there is an increase in methylation at the cg00862770 locus. Among those who have diabetes and exercise, no difference in methylation at the cg00862770 locus was observed.

Overall, variations in the FKBP5 gene and its methylation levels may lead to various mental illnesses and health issues. Further research will help us better understand these associations and their impact on disease development.

## Figures and Tables

**Table 1:** Basic characteristics.

	**Without diabetes**	**With diabetes**	**p** ** value**
	**(** **n** = 998**)**	**(** **n** = 105**)**	
Cg22363520	0.9142 ± 0.000386	0.9147 ± 0.00119	0.6872
Cg00862770	0.0507 ± 0.000349	0.0512 ± 0.00108	0.6569
Exercise			0.0023
No	573 (5741)	44 (41.90)	
Yes	425 (42.59)	61 (58.10)	
Sex			0.0103
Female	502 (50.30)	39 (37.14)	
Male	496 (49.70)	66 (62.86)	
Age	48.9609 ± 0.3489	55.9905 ± 0.9296	< 0.0001
Body fat rate	27.0306 ± 0.2339	28.2219 ± 0.8290	0.1692
Waist–hip ratio	0.8599 ± 0.00205	0.9208 ± 0.00601	< 0.0001
Smoking			0.1139
Never	757 (75.85)	70 (66.67)	
Quit	136 (13.63)	19 (18.10)	
Current	105 (10.52)	16 (15.24)	
Second hand smoking			0.3496
No	886 (88.78)	90 (85.71)	
Yes	112 (11.22)	15 (14.29)	
Drinking			0.0009
Never	902 (90.38)	90 (85.71)	
Quit	27 (2.71)	10 (9.52)	
Current	69 (6.91)	5 (4.76)	

*Note:* Data are presented as mean ± SE or numbers (percentages).

**Table 2:** The interaction between diabetes and exercise on FKBP5 (cg22363520 and cg00862770) using multiple linear regression.

	**cg22363520**		**cg00862770**	
	**β**	**p** ** value**	**β**	**p** ** value**
Diabetes (ref: no)				
Yes	0.00125	0.3202	0.00047263	0.6875
Exercise (ref: no)				
Yes	−0.00144	0.0590	−0.00020538	0.7736
Sex (ref: female)				
Male	0.00055891	0.6211	0.00131	0.2135
Age	0.00014311	0.0001	−0.00011592	0.0008
Body fat rate	0.00008671	0.1949	−0.00003774	0.5460
Waist–hip ratio	−0.01435	0.0387	0.01058	0.1026
Smoking (ref: never)				
Quit	−0.00138	0.2121	−0.00015905	0.8781
Current	−0.00196	0.1168	0.00066013	0.5720
Second hand smoking (ref: no)				
Yes	0.00061988	0.5856	−0.00130	0.2210
Drinking (ref: never)				
Quit	0.00050293	0.8034	−0.00136	0.4707
Current	0.00124	0.4007	0.00136	0.3244
Interaction	*p* value = 0.0336		*p* value = 0.0021	

*Note: *Interaction = diabetes∗exercise.

**Table 3:** Stratified by diabetes.

	**cg22363520**				**cg00862770**			
	**Without diabetes**		**With diabetes**		**Without diabetes**		**With diabetes**	
	**β**	**p** ** value**	**β**	**p** ** value**	**β**	**p** ** value**	**β**	**p** ** value**
Exercise (ref: no)								
Yes	−0.00195	0.0157	0.00312	0.2207	0.00041955	0.5766	−0.00611	0.0081
Sex (ref: female)								
Male	0.00087089	0.4582	−0.00261	0.5528	0.00110	0.3166	0.00345	0.3799
Age	0.00014571	0.0002	0.00009489	0.5321	−0.00012251	0.0007	0.00002095	0.8769
Body fat rate	0.00009380	0.1812	0.00004286	0.8604	−0.00005128	0.4339	0.00008682	0.6896
Waist–hip ratio	−0.01251	0.0847	−0.03080	0.2348	0.00821	0.2254	0.02283	0.3226
Smoking (ref: never)								
Quit	−0.00152	0.1962	−0.00003528	0.9920	0.00015696	0.8866	−0.00367	0.2417
Current	−0.00223	0.0964	−0.00117	0.7483	0.00135	0.2815	−0.00317	0.3309
Second hand smoking (ref: no)								
Yes	0.00077131	0.5239	−0.00187	0.6047	−0.00190	0.0923	0.00440	0.1733
Drinking (ref: never)								
Quit	0.00226	0.3338	−0.00437	0.3016	0.00000887	0.9968	−0.00519	0.1695
Current	0.00132	0.3883	−0.00012968	0.9822	0.00138	0.3354	0.00248	0.6317

**Table 4:** Stratified by exercise.

	**cg22363520**				**cg00862770**			
	**No exercise**		**Exercise**		**No exercise**		**Exercise**	
	**β**	**p** ** value**	**β**	**p** ** value**	**β**	**p** ** value**	**β**	**p** ** value**
Diabetes (ref: no)								
Yes	−0.00180	0.3511	0.00377	0.0241	0.00456	0.0076	−0.00272	0.0960
Sex (ref: female)								
Male	0.00110	0.4707	0.00016516	0.9249	0.00185	0.1700	0.00010280	0.9522
Age	0.00015064	0.0024	0.00012955	0.0226	−0.00013938	0.0015	−0.00007918	0.1539
Body fat rate	0.00009742	0.2758	0.00007120	0.4942	0.00002251	0.7756	−0.00012184	0.2324
Waist-hip ratio	−0.01479	0.1343	−0.01307	0.1840	0.00479	0.5825	0.01371	0.1548
Smoking (ref: never)								
Quit	−0.00263	0.0919	−0.00007265	0.9639	0.00144	0.2965	−0.00136	0.3875
Current	−0.00263	0.0925	−0.00080968	0.7033	0.00201	0.1457	−0.00160	0.4418
Second hand smoking (ref: no)								
Yes	0.00114	0.4117	−0.00063913	0.7532	−0.00315	0.0105	0.00216	0.2786
Drinking (ref: never)								
Quit	0.00295	0.3193	−0.00249	0.3732	0.00068787	0.7926	−0.00148	0.5881
Current	0.00087910	0.6469	0.00183	0.4309	0.00240	0.1579	−0.0001453	0.9491

## Data Availability

The data that support the findings of this study are available on request from the corresponding authors. The data are not publicly available due to privacy or ethical restrictions.

## Nomenclature

FKBP5: FK506 binding protein 5
IDF: International Diabetes Federation
DM: diabetes mellitus
GC: glucocorticoid
SAT: subcutaneous adipose tissue
PTSD: posttraumatic stress disorder
AD: Alzheimer's disease
T2D: Type 2 diabetes

## Supporting Information

Supporting Information Additional supporting information can be found online in the Supporting Information section. We provide data stratified by gender and exercise status in Table S1-1 and S1-2, including body fat percentage and waist-to-hip ratio. Table S1-1 provides a stratified analysis of participants' baseline characteristics by gender, including demographic information (e.g., age and BMI), behavioral traits (smoking and alcohol consumption), diabetes status, methylation levels at cg22363520 and cg00862770 CpG sites, and other potential influencing factors. Table S1-2 provides a stratified analysis of participants' baseline characteristics based on exercise status, including demographic information (e.g., age and BMI), behavioral traits (smoking and alcohol consumption), diabetes status, methylation levels at cg22363520 and cg00862770 CpG sites, and other potential influencing factors. Finally, Figures S1-1, S1-2, and S1-3 summarize the mean methylation levels of FKBP5 at cg22363520 and cg00862770, presenting methylation data stratified by diabetes status, gender, and exercise status. Figure S1-4 present a summarize model illustrating the relationship and interaction between exercise and diabetes concerning FKBP5 gene methylation levels at the two specific CpG sites (cg22363520 and cg00862770). The bars show the change in methylation levels (*β* values) for participants with and without diabetes, as well as those with and without exercise habits. The first graph displays the effect on the cg22363520 methylation site, where exercise appears to reduce methylation in participants without diabetes, but not in those with diabetes. The second graph demonstrates the changes in methylation at the cg00862770 site, showing a more significant reduction in methylation levels for diabetic participants who exercise.
