# Supplementary material for: Delving Into the Interaction Between Exercise and Diabetes on Methylation of the FKBP5 Gene
Source: J Diabetes Res. 2025 Feb 19;2025:1162708. doi: 10.1155/jdr/1162708 (PMC11865466; doi:10.1155/jdr/1162708)
Supplement: Supporting Information — Additional supporting information can be found online in the Supporting Information section. We provide data stratified by gender and exercise status in Table S1-1 and S1-2, including body fat percentage and waist-to-hip ratio. Table S1-1 provides a stratified analysis of participants' baseline characteristics by gender, including demographic information (e.g., age and BMI), behavioral traits (smoking and alcohol consumption), diabetes status, methylation levels at cg22363520 and cg00862770 CpG sites, and other potential influencing factors. Table S1-2 provides a stratified analysis of participants' baseline characteristics based on exercise status, including demographic information (e.g., age and BMI), behavioral traits (smoking and alcohol consumption), diabetes status, methylation levels at cg22363520 and cg00862770 CpG sites, and other potential influencing factors. Finally, Figures S1-1, S1-2, and S1-3 summarize the mean methylation levels of FKBP5 at cg22363520 and cg00862770, presenting methylation data stratified by diabetes status, gender, and exercise status. Figure S1-4 present a summarize model illustrating the relationship and interaction between exercise and diabetes concerning FKBP5 gene methylation levels at the two specific CpG sites (cg22363520 and cg00862770). The bars show the change in methylation levels (β values) for participants with and without diabetes, as well as those with and without exercise habits. The first graph displays the effect on the cg22363520 methylation site, where exercise appears to reduce methylation in participants without diabetes, but not in those with diabetes. The second graph demonstrates the changes in methylation at the cg00862770 site, showing a more significant reduction in methylation levels for diabetic participants who exercise. [file 1162708.f1.docx]

**Table 1-1. Basic characteristics. (stratified by gender)**

|  | **Female**  (n=541) | **Male**  (n=562) | **p-value** |
| --- | --- | --- | --- |
| **cg22363520** | 0.9150±0.000538 | 0.9136±0.000500 | 0.0618 |
| **cg00862770** | 0.0496±0.000473 | 0.0519±0.000461 | 0.0005 |
| **Exercise** |  |  | 0.3710 |
| No | 310(57.30) | 307(54.63) |  |
| Yes | 231(42.70) | 255(45.37) |  |
| **Diabetes** |  |  | 0.0103 |
| No | 502(92.79) | 496(88.26) |  |
| Yes | 39(7.21) | 66(11.74) |  |
| **Age** | 49.3087±0.4599 | 49.9395±0.4824 | 0.3447 |
| **Body fat rate** | 31.6778±0.2851 | 22.7795±0.2289 | <0.0001 |
| **Waist-hip ratio** | 0.8397±0.00295 | 0.8907±0.00230 | <0.0001 |
| **Smoking** |  |  | <0.0001 |
| Never | 507(93.72) | 320(56.94) |  |
| Quit | 19(3.51) | 136(24.20) |  |
| Current | 15(2.77) | 106(18.86) |  |
| **Second hand smoking** |  |  | 0.4181 |
| No | 483(89.28) | 493(87.72) |  |
| Yes | 58(10.72) | 69(12.28) |  |
| **Drinking** |  |  | <0.001 |
| Never | 527(97.41) | 465(82.74) |  |
| Quit | 6(1.11) | 31(5.52) |  |
| Current | 8(1.48) | 66(11.74) |  |

This table provides a stratified analysis of participants' baseline characteristics by gender, including demographic information (e.g., age, BMI), behavioral traits (smoking and alcohol consumption), diabetes status, methylation levels at cg22363520 and cg00862770 CpG sites, and other potential influencing factors.

**Table 1-2. Basic characteristics. (stratified by exercise)**

|  | **No exercise**  (n=617) | **Exercise**  (n=486) | **p-value** |
| --- | --- | --- | --- |
| **cg22363520** | 0.9149±0.000490 | 0.9134±0.000553 | 0.0404 |
| **cg00862770** | 0.0510±0.000427 | 0.0505±0.000523 | 0.4054 |
| **Sex** |  |  | 0.3710 |
| Female | 310(50.24) | 231(47.53) |  |
| Male | 307(49.76) | 255(52.47) |  |
| **Diabetes** |  |  | 0.0023 |
| No | 573(92.87) | 425(87.45) |  |
| Yes | 44(7.13) | 61(12.55) |  |
| **Age** | 46.0956±0.4214 | 54.1173±0.4617 | <0.0001 |
| **Body fat rate** | 27.4833±0.3036 | 26.7132±0.3377 | 0.0907 |
| **Waist-hip ratio** | 0.8617±0.00272 | 0.8708±0.00298 | 0.0245 |
| **Smoking** |  |  | 0.0053 |
| Never | 460(74.55) | 367(75.51) |  |
| Quit | 75(12.16) | 80(16.46) |  |
| Current | 82(13.29) | 39(8.02) |  |
| **Second hand smoking** |  |  | 0.0003 |
| No | 527(85.41) | 449(92.39) |  |
| Yes | 90(14.59) | 37(7.61) |  |
| **Drinking** |  |  | 0.3309 |
| Never | 555(89.95) | 437(89.92) |  |
| Quit | 17(2.76) | 20(4.12) |  |
| Current | 45(7.29) | 29(5.97) |  |

This table provides a stratified analysis of participants' baseline characteristics based on exercise status, including demographic information (e.g., age, BMI), behavioral traits (smoking and alcohol consumption), diabetes status, methylation levels at cg22363520 and cg00862770 CpG sites, and other potential influencing factors.

Figure 1-1. Mean methylation level. (stratified by diabetes)

This figure presents the mean methylation levels at cg22363520 and cg00862770 CpG sites for diabetic and non-diabetic participants.

Figure 1-2. Mean methylation level. (stratified by gender)

This figure shows the mean methylation levels at cg22363520 and cg00862770 CpG sites for male and female participants.

Figure 1-3. Mean methylation level. (stratified by exercise)

This figure illustrates the mean methylation levels at cg22363520 and cg00862770 CpG sites for individuals who exercise regularly and those who do not.

Figure 1-4. Summarize model

The figure above presents a mechanistic model illustrating the relationship and interaction between exercise and diabetes on the methylation levels of the FKBP5 gene at two specific CpG sites: cg22363520 and cg00862770. The bars show the change in methylation levels (β-values) for participants with and without diabetes, as well as those with and without exercise habits.

The first graph displays the effect on the cg22363520 methylation site, where exercise appears to reduce methylation in participants without diabetes, but not in those with diabetes.

The second graph demonstrates the changes in methylation at the cg00862770 site, showing a more significant reduction in methylation levels for diabetic participants who exercise.
